# 

**Published:** 1888-08

**Authors:** 


### CONTENTS.

COMMUNICATIONS.

Cancrum or Scirrhus Gangrenousa of the Inferior Maxillary........... 369
Development of the Teeth................ ........................................ 373
Illinois State Dental Society ................................................ 377
Kentucky State Dental Society............................................... 393

SELECTIONS.

Electricity in Surgery.......................................................... 398
Three Cases of Scurvy.......................................................... 401
Philadelphia County Medical Society....................................... 408
A Cheap Battery................................................................. 411
A Misrepresentation Corrected.................. ........................ ...... 412
The Transplantation of the Mucous Membrane.......................... 412
Transactions of the Ninth International Medical Congress............ 413
Corrosive Sublimate for Hardening the Brain for Anatomical Study 413
A New Cure for Stuttering..................................................... 413
Salmagundi ........................................................................ 413
Correspondence.................................................................. 415—416

EDITORIAL.

National Association of Dental Examiners................................. 417
Obituary.................... ..................... ................................. 417
Annual Meeting of the Dental Faculties Association.................... 418
Information......................................................................... 419
California State Dental Society............................................... 420
American Dental Association.................................................. 420

